# Blockade of deubiquitinating enzyme PSMD14 overcomes chemoresistance in head and neck squamous cell carcinoma by antagonizing E2F1/Akt/SOX2-mediated stemness

**DOI:** 10.7150/thno.48375

**Published:** 2021-01-01

**Authors:** Chao Jing, Yuansheng Duan, Mengqian Zhou, Kai Yue, Shanshan Zhuo, Xingchen Li, Dandan Liu, Beibei Ye, Qingchuan Lai, Linqi Li, Xiaofeng Yao, Hui Wei, Wenchao Zhang, Yansheng Wu, Xudong Wang

**Affiliations:** 1 Department of Maxillofacial and Otorhinolaryngological Oncology, Tianjin Medical University Cancer Institute and Hospital, Key Laboratory of Cancer Prevention and Therapy, Tianjin Cancer Institute, National Clinical Research Center of Cancer, Tianjin 300060, China.; 2 Department of Ear, Nose and Throat, Tianjin Hospital, Tianjin 300211, China.; 3 Department of Radiotherapy, Tianjin Medical University Cancer Institute and Hospital, Key Laboratory of Cancer Prevention and Therapy, Tianjin Cancer Institute, National Clinical Research Center of Cancer, Tianjin 300060, China.

**Keywords:** Head and neck squamous cell carcinoma, PSMD14, E2F1, Chemoresistance, Thiolutin

## Abstract

Increasing evidence reveals a close relationship between deubiquitinating enzymes (DUBs) and cancer progression. In this study, we attempted to identify the roles and mechanisms of critical DUBs in head and neck squamous cell carcinoma (HNSCC).

**Methods:** Bioinformatics analysis was performed to screen differentially expressed novel DUBs in HNSCC. Immunohistochemistry assay was used to measure the expression of DUB PSMD14 in HNSCC specimens and adjacent normal tissues. The level of PSMD14 in HNSCC tumorigenesis was investigated using a 4-NQO-induced murine HNSCC model. The function of PSMD14 was determined through loss-of-function assays. Chromatin immunoprecipitation, immunoprecipitation and* in vivo* ubiquitination assay were conducted to explore the potential mechanism of PSMD14. The anti-tumor activity of PSMD14 inhibitor Thiolutin was assessed by *in vitro* and *in vivo* experiments.

**Results:** We identified PSMD14 as one of significantly upregulated DUBs in HNSCC tissues. Aberrant expression of PSMD14 was associated with tumorigenesis and malignant progression of HNSCC and further indicated poor prognosis. The results of *in vitro* and *in vivo* experiments demonstrated PSMD14 depletion significantly undermined HNSCC growth, chemoresistance and stemness. Mechanically, PSMD14 inhibited the ubiquitination and degradation of E2F1 to improve the activation of Akt pathway and the transcription of SOX2. Furthermore, PSMD14 inhibitor Thiolutin exhibited a potent anti-tumor effect on HNSCC* in vivo* and *in vitro* by impairing DUB activity of PSMD14.

**Conclusion:** Our findings demonstrate the role and mechanism of PSMD14 in HNSCC, and provide a novel and promising target for diagnosis and clinical therapy of HNSCC.

## Introduction

Head and neck cancer (HNC) ranks the 7th most common cancer, with more than 700,000 new cases in 2018 worldwide 1. As the dominant histological subtype, head and neck squamous cell carcinoma (HNSCC) accounts for approximately 90% of HNC diagnosed all over the world, caused by tobacco, alcohol as well as human papilloma virus (HPV) infection 2. Despite therapeutic advancements, the 5-year overall survival rate of HNSCC patients remains about 50%. Cisplatin-based chemotherapy is the standard treatment for patients with HNSCC. However, either intrinsic or acquired chemoresistance contributes to treatment failure, loco-regional recurrence and even unfavorable prognosis. Novel strategies for HNSCC treatment, therefore, are urgently needed.

Chemoresistance results from multiple mechanism including tumor cell extrinsic factors, such as hypoxia environment 3 and the tumor stroma 4, as well as intrinsic factors including alteration in intracellular drug concentrations 5, efficient DNA repair systems 6 and inhibition of apoptosis 7. In addition, the tumor cells become highly tumorigenic and chemoresistant when they have the characteristics of stem cell. For instance, carcinoma-associated fibroblasts (CAFs) as a major component of the tumor stroma could sustain cancer stemness to promote cancer formation and chemoresistance 8. In contrast, the tumor suppressor ARID1A inhibits cancer stemness of squamous cell carcinoma by antagonizing pRb/E2F1/c-Myc pathway, ultimately impairs resistance to chemotherapy 9. Unfortunately, these discoveries are just the tip of the iceberg and are not fully translated into clinical practice. Thus, the potential mechanisms merit further exploration to improve chemotherapy.

Ubiquitin proteasome system (UPS), which mediates the degradation of more than 80% proteins in eukaryotic cells, plays a critical role in orchestrating the function and stabilization of proteins 10. Of note, the ubiquitination of substrates, similar to other posttranslational modifications, is a dynamic and reversible process counterbalanced by deubiquitinating enzymes (DUBs) that cleave ubiquitin from the substrates, modify ubiquitin chains and process ubiquitin precursors. DUBs are classified into 7 families including USPs (ubiquitin-specific proteases), UCHs (ubiquitin carboxy-terminal hydrolases), OTUs (ovarian tumor proteases), MJDs (Machado-Josephin domain-containing proteases), MINDYs (motif-interacting with ubiquitin-containing novel DUB family), JAMMs (the JAB1, MPN, MOV34 family) and ZUP1 (zinc finger containing ubiquitin peptidase 1) 11, 12. Previous studies have revealed that DUBs are implicated in tumorigenesis 13, 14 and tumor progression, such as proliferation 15, apoptosis 16, metastasis 17, immunosuppression 18 and chemoresistance 19, 20. Given their importance, DUBs are expected to act as potential targets for cancer therapy.

Here, we identified ten elevated DUBs including PSMD14 in HNSCC using bioinformatics analysis. The results of immunohistochemistry showed that PSMD14 expression correlated positively with clinical stage, T stage and recurrence. Importantly, Kaplan-Meier analysis uncovered that higher expression of PSMD14 indicated unfavorable prognosis of patients with HNSCC. Through 4-NQO-induced HNSCC model, we found an increase of PSMD14 in the process of HNSCC tumorigenesis. The* in vitro* and *in vivo* assays suggested that PSMD14 depletion significantly impaired tumor growth, chemoresistance in HNSCC by antagonizing E2F1/Akt/SOX2 axis-mediated cancer cell stemness. Moreover, we verified that targeting PSMD14 by Thiolutin (THL), a small-molecule inhibitor of PSMD14, dramatically sensitized HNSCC cells to cisplatin *in vitro* and *in vivo*. Our study identified PSMD14 as a novel DUB that play a critical role in the chemoresistance of HNSCC and provided a promising therapeutic approach involving the administration of THL to overcome chemoresistance in HNSCC.

## Materials and Methods

### Bioinformatics analysis

90 potential DUBs were analyzed by using public databases. The online software GEPIA (gepia.cancer-pku.cn) was provided to analyze TCGA (The Cancer Genome Atlas) database, and GEO2R was used to identify gene expression in GEO (Gene Expression Omnibus) datasets GSE13601, GSE33205, GSE37991 and GSE30784.

### Antibodies and reagents

The following antibodies are used for immunoblotting (IB), immunoprecipitation (IP), immunohistochemistry (IHC), immunofluorescence (IF) and Chromatin immunoprecipitation (ChIP) assay in this study: PSMD14, Proteintech (Rosemont, IL, USA), 12059-1-AP (IB, 1:1000); PSMD14, Sigma-Aldrich (St. Louis, MO, USA), HPA002114 (IHC, 1:400); PSMD14, Santa Cruz (Dallas, Texas, USA), sc-100464 (IF, 1:200); PARP, Cell Signaling Technology (Danvers, MA, USA), #9542 (IB, 1:1000); Caspase-3, Abcam (Cambridge, UK), ab32351 (IB, 1:1000); Cleaved Caspase-3, Cell Signaling Technology, #9661 (IHC, 1:400); SOX2, Proteintech, 11064-1-AP (IB, 1:1000; IF, 1:200; IHC, 1:200); Akt, Cell Signaling Technology, #4685 (IB, 1:1000); Phospho-Akt (Ser473), Cell Signaling Technology, #4060 (IB, 1:1000); E2F1, Abcam, ab179445 (IB, 1:1000; IP, 1:100; IHC, 1:200; ChIP, 1:500); Ubiquitin, Cell Signaling Technology, #3936 (IB, 1:1000); Ki67, Cell Signaling Technology, #9449 (IHC, 1:500); Erk1/2, Cell Signaling Technology, #4695 (IB, 1:1000); Phospho-Erk1/2 (Thr202/Tyr204), Cell Signaling Technology, #4370 (IB, 1:1000); Stat3, Cell Signaling Technology, #9139 (IB, 1:1000); Phospho-Stat3 (Tyr705), Cell Signaling Technology, #9145 (IB, 1:1000); GAPDH, Santa Cruz, sc-365062 (IB, 1:5000) and Normal Rabbit IgG, Cell Signaling Technology, #2729 (IP and ChIP). 4-Nitroquinoline 1-oxide (4-NQO) was obtained from Sigma-Aldrich, MG132, CHX and MK2206 were purchased from Selleck (Shanghai, China), and Thiolutin (THL) was purchased from Tocris Bioscience (Avonmouth, Bristol, UK).

### HNSCC specimens and immunohistochemical analysis

88 cases of HNSCC tissue specimens were collected from patients underwent surgery in Tianjin Medical University Cancer Hospital. All experiments performed on tissue samples were approved by the ethical committee of Tianjin Medical University Cancer Institute and Hospital.

Formalin-fixed, paraffin-embedded HNSCC tissues were deparaffinized, rehydrated and incubated with 0.3% H_2_O_2_ for 30 minutes at room temperature to block endogenous peroxidase. After antigen retrieval, the sections were blocked with diluted goat serum for 30 min and then incubated with the primary antibodies overnight at 4 °C. Stained by using the avidin-biotin immunoperoxidase method, mounted specimens were visualized and analyzed by ImageScope software (Leica Biosystems, Nussloch, Germany).

### Animal experiments

All animal protocols were approved by the Animal Care and Use Committee of Tianjin Medical University Cancer Institute and Hospital. For 4-NQO-induced HNSCC animal model, 6-week-old immunocompromised mice (BALB/C nude mice) were given with water containing 4-NQO (50 μg/mL) for 16 weeks and then treated with normal drinking water for another 12 weeks. Tongues from the subjects were harvested at week 16, 24 and 28 for further histopathological analysis.

For *in vivo* growth assay, 1×10^6^ HNSCC cells were injected subcutaneously into the flanks of 6-week-old BALB/c nude mice. To minimize variations among individuals, the negative control and treated HNSCC SCC15 cells were implanted in the same mice. The tumors were collected at 4 weeks and weighed to assess the proliferation capacity of tumor cells between two groups.

For tumor formation assay, SCC15 cells (1×10^3^, 1×10^4^, 1×10^5^, 1×10^6^) premixed with Matrigel (Corning, NY, USA) at a ratio of 1:1 were injected subcutaneously into 6-week-old BALB/c nude mice (7 mice per group). After 4 weeks, tumor formation was recorded.

For *in vivo* chemosensitivity test, 1×10^6^ HNSCC stable clones (labeled as Vehicle, PSMD14, PSMD14+shE2F1) were injected subcutaneously into 6-week-old BALB/c nude mice. CDDP (2.5 mg/kg) was administered three times a week until sacrifice.

For *in vivo* treatment assay, 20 BALB/c nude mice (6 weeks of age) were injected subcutaneously with 1×10^6^ SCC15 cells. Until tumor establishment, the mice were randomly allocated to 4 groups (5 mice per group) and administered with saline, THL (0.75 mg/kg), cisplatin (CDDP, 2.5 mg/kg) or CDDP (2.5 mg/kg) combined with THL (0.75 mg/kg) every 3 days, respectively. All the animals were euthanized and the tumors were harvested, fixed and embedded by paraffin for IHC detection.

### Cell culture

HNSCC cell lines CAL27, SCC15 and SCC25 were obtained from the American Type Culture Collection (ATCC, Manassas, VA, USA). The UM1 cell line was a generous gift from Prof. Jinsong Hou (Sun Yat-sen University, Guangzhou, China). The Hep-2 and TSCCA cell lines were purchased from the Institute of Basic Medical Sciences, Chinese Academy of Medical Sciences, and the Tb3.1 cell line was a gift from Professor Chenping Zhang (Shanghai Jiaotong University, Shanghai, China). All cell lines were authenticated by short tandem repeat (STR) genotyping. The cell lines SCC15, SCC25 and UM1 were maintained in DMEM/Ham's F12 supplemented with 10% FBS at 37°C in a humidified atmosphere of 5% CO_2_. All cells were checked for *Mycoplasma* contamination before experiments.

To establish chemoresistant cell sublines SCC15/CDDP-R and UM1/CDDP-R, the cells were treated with increasing doses of CDDP every two to three weeks. Until the 50% inhibitory concentration (IC50) for CDDP reached 5 times as many as that in the parental cells, the acquired chemoresistant cell subclones were established. Parental cells were passaged alongside the cells under CDDP selection pressure.

### Transfection and transduction

Small interfering RNAs (siRNAs) targeting PSMD14, E2F1 and negative control were purchased from RIBOBIO (Guangzhou, China). The siRNAs sequences were shown in Table S1. The shRNA sequences targeting PSMD14 (shPSMD14-1), E2F1 and SOX2 were 5'-CAAGCCATCTATCCAGGCATT-3', 5'-CGCTATGAGACCTCACTGAAT-3' and 5'-CAGCTCGCAGACCTACATGAA-3', which were cloned into the pSIH1-H1-puro vector. Transfection, lentivirus package and transduction were performed as previously described 21. The HNSCC cells expressing shPSMD14-1 were labeled as shPSMD14 and used in the *in vitro* and *in vivo* assay.

### Immunoblotting

Cells were lysed in RIPA buffer supplemented with protease and phosphatase inhibitors (Roche, Basel, Switzerland) and centrifuged at 14,000×g for 15 min (4°C) to collect the supernatants. The total protein concentrations were measured by a BCA protein assay kit (Thermo Fisher Scientific, Waltham, MA, USA). After denaturation and gel electrophoresis, the proteins were transferred onto PVDF membranes (Merck Millipore, Billerica, MA, USA). The membranes were blocked with 5% non-fat milk and then incubated with primary antibodies at 4°C overnight. The images were obtained by ImageQuant LAS4000 System (GE, Fairfeld, Connecticut, USA).

### Quantitative real-time PCR (qPCR)

Total RNA from cells was extracted by using TRIzol reagent (Invitrogen, Waltham, MA, USA) according to standard instructions and then reversely transcribed to cDNA using PrimeScript™ RT Master Mix (TaKaRa, Shiga, Japan). The expression analysis was performed on Q5 real-time PCR system (Applied Biosystems, Foster City, CA, USA) by using SYBR Premix Ex Taq^TM^ II (TaKaRa). GAPDH was served as a loading control to normalize the expression levels of target genes. The relative abundance of genes was determined by using 2^-ΔΔCt^ method. The primers for GAPDH and target genes were listed in Table S2.

### Immunofluorescence

HNSCC cells were plated on 18-mm cover glasses until they adhered to the surface. The cells were fixed and permeabilized with 0.1% Triton X-100. Then, they were blocked by 5% bovine serum albumin (BSA) and incubated with primary antibodies against PSMD14 and SOX2 at 4°C overnight. The proteins were visualized by incubation with anti-rabbit IgG conjugated to Alexa Fluor 488 or anti-mouse IgG conjugated to Alexa Fluor 594 (Cell Signaling Technology) for 1 hour at room temperature, and the nuclei were stained with 4,6-diamidino-2-phenylindole (DAPI, Thermo Fisher Scientific) for another 10 min. All images were obtained by Iamger.Z2 (Zeiss, Oberkochen, Germany).

### MTT assay

For cell growth detection, HNSCC cells (1500 cells/well) were seeded into 96-well plates with complete medium. For assessing cell viability to drugs, HNSCC cells (5000 cells/well) were planted into 96-well plates until adherence, and then exposed to CDDP or THL at various concentrations for 24 hours. At the designated time, MTT assay was performed as previously described 22, and IC50 was calculated using GraphPad Prism 6 (La Jolla, USA).

### Clonogenicity assay

HNSCC cells were seeded into a 6-well plate (500 cells per well) and cultured for 10-14 days. Then, the cells were washed twice with PBS and fixed with methanol. The colonies were stained with 0.1% crystal violet and counted (> 50 cells).

### Flow cytometry

Apoptosis assay was performed using Annexin V/PI Apoptosis Detection kit (BD, Franklin Lakes, NJ, USA) according to the manufacturer's instruction. Apoptosis of treated cells was evaluated on the same FACS Canto II (BD).

### Sphere formation assay

500 HNSCC cells were seeded into 6-well ultra-low cluster plates and were cultured in DMEM/F12 serum-free medium supplemented with 2% B27 (Invitrogen), 20 ng/ml EGF (PeproTech, Rocky Hill, NJ, USA), 20 ng/ml bFGF (PeproTech), 0.4% BSA and 5 μg/ml insulin for 10-14 days. The spheres (> 75 μm in diameter) in the whole well were counted using an inverted microscope (DMI6000B, Leica).

### *In vivo* ubiquitination assays

To detect endogenous ubiquitination of E2F1, PSMD14 depletion cells (shPSMD14) and negative control (shNC) were incubated with MG132 (10 μM) for 8 hours and then lysed by RIPA buffer. Proteins in the cell lysate were immunoprecipitated to isolate ubiquitinated E2F1 with an anti-E2F1 antibody and the endogenous ubiquitin chains on E2F1 were detected through immunoblotting assay with an antibody against ubiquitin.

### Chromatin immunoprecipitation (ChIP) assay

ChIP assay was conducted using EZ-Magna ChIP™ A/G Chromatin Immunoprecipitation Kit (Merck Millipore) according to the manufacturer's protocol. Interactions of E2F1 with the promoter of SOX2 were assessed by PCR amplification. The primers were shown in Table S3.

### Ubiquitin-AMC assays

Recombinant JAMM DUBs (20 nM) were incubated with dimethyl sulfoxide (DMSO) or THL in assay buffer (40 mm Tris/HCl pH 7.4; 5% glycerol; 0.005% Tween-20; 1 mm dithiothreitol; 0.05 mg/ml ovalbumin) at 37 °C for 30 min, and then Ub-AMC (500 nM, R&D systems, Minnesota, USA) was added for another 20 min, followed by detecting fluorescence intensity.

### Statistical analysis

All assays other than IHC and animal experiments were repeated at least three times. Data were presented as mean ± standard deviation and analyzed by using unpaired or paired Student's* t*-test unless stated particularly. A value of *P* < 0.05 was considered statistically significant. Graphs were illustrated by GraphPad Prism 6, in which *, **, *** and **** indicated *P* < 0.05, *P* < 0.01, *P* < 0.001, *P* < 0.0001, respectively.

## Results

### Aberrant expression of PSMD14 indicates poor prognosis of HNSCC patients

**Figure 1:**
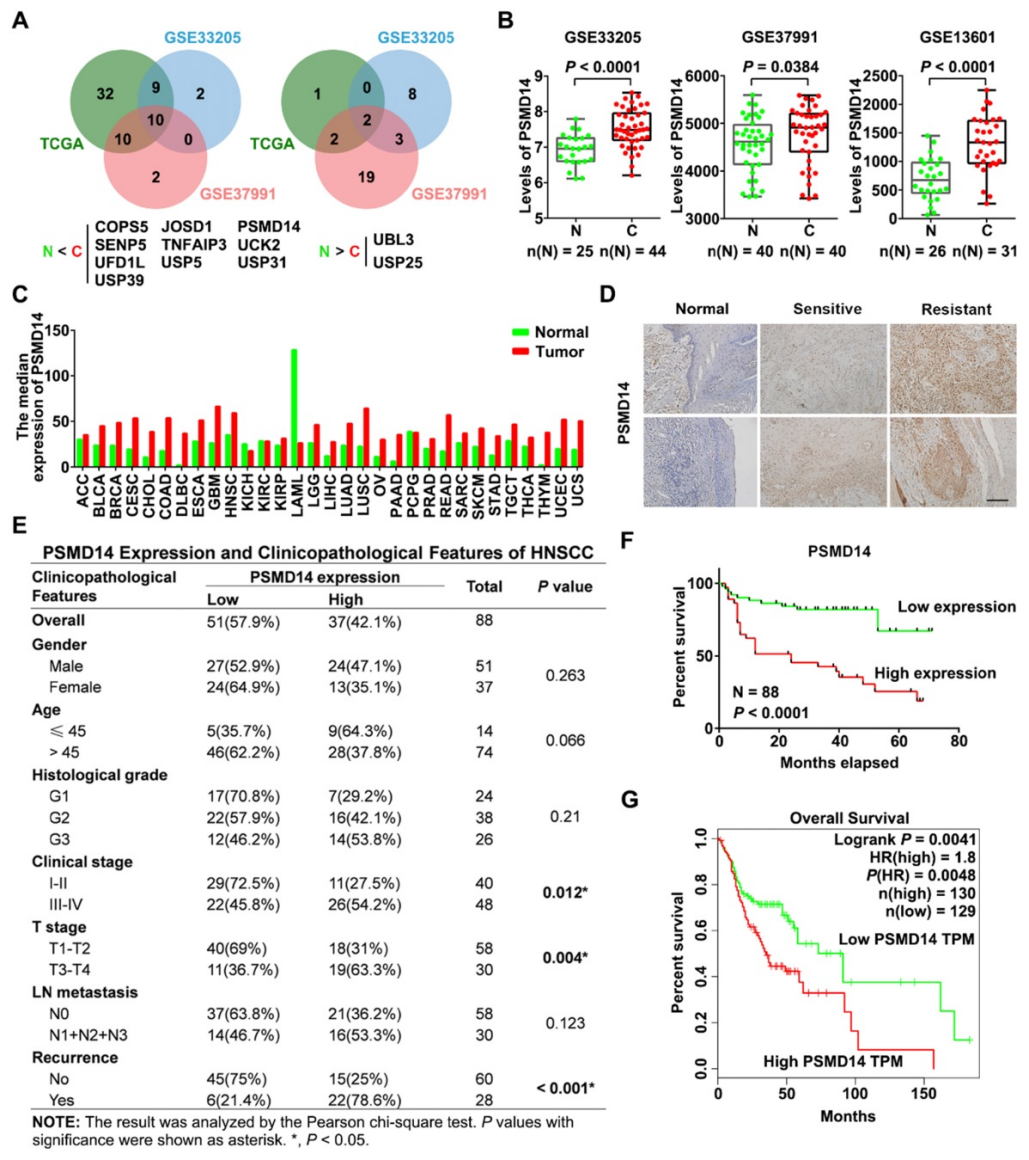
** Elevated PSMD14 predicts poor overall survival in HNSCC. (A)** The Venn diagrams identified differentially expressed DUBs in HNSCC. N, normal tissue. C, cancerous tissue. **(B)** PSMD14 was overexpressed in HNSCC compared to adjacent normal tissues in three independent cohorts (GSE33205, GSE37991, GSE13601). N, normal tissue. C, cancerous tissue. Data, mean ± SD.** (C)** The analysis of TCGA database showed that the level of PSMD14 was increased in a broad spectrum of human cancers including HNSCC. **(D)** The expression of PSMD14 in adjacent normal tissues and HNSCC specimens (sensitive/resistant samples) was measured using IHC assay. Scale bar, 100 μm. **(E)** The correlation between PSMD14 expression and clinicopathological features of HNSCC. 88 cases of HNSCC specimens were analyzed.** (F)** Kaplan-Meier survival curve showed that PSMD14 overexpression indicated poor prognosis in HNSCC. **(G)** TCGA data confirmed that Higher *PSMD14* level predicted shorter overall survival of HNSCC patients. HR, hazard rate.

To identify essential DUBs expressed in HNSCC tissues, we first analyzed the level of 90 DUBs from TCGA database and two GEO datasets concerning the expression profiles in HNSCC tissues (GSE33205, GSE37991). We found that many DUBs were differentially expressed between HNSCC and adjacent normal tissues in each cohort, and then we identified 10 significantly upregulated DUBs in HNSCC using Venn diagram (Figure 1A). Among them, PSMD14 was one of the most increased DUBs in HNSCC tissues (further verified by analyzing another GEO dataset GSE13601) and cell lines (Figure 1B and Figure S1A). Besides, bioinformatics analysis showed that *PSMD14* was dramatically elevated in many other cancers (Figure 1C), suggesting that PSMD14 may be involved in the malignant progression of multiple cancers including HNSCC. Next, to investigate the correlation between PSMD14 level and clinicopathological features, we measured the expression of PSMD14 in 88 HNSCC tissues using IHC staining. As shown in Figure 1D, PSMD14 expression of HNSCC tissues was much higher than that of normal tissues. Particularly, PSMD14 was expressed more highly in chemoresistant tissues (Figure 1D), indicating a potential role of PSMD14 in HNSCC chemoresistance. Besides, PSMD14 expression correlated positively with clinical stage, T stage and recurrence (Figure 1E). Furthermore, Kaplan-Meier analysis demonstrated that HNSCC patients with higher PSMD14 expression showed a worse outcome (Figure 1F), which was reconfirmed by GEPIA-analyzed overall survival plot of HNSCC (Figure 1G). Taken together, these results suggest that DUB PSMD14 is aberrantly expressed in HNSCC and predicts unfavorable prognosis.

### PSMD14 promotes tumor initiation in HNSCC

**Figure 2:**
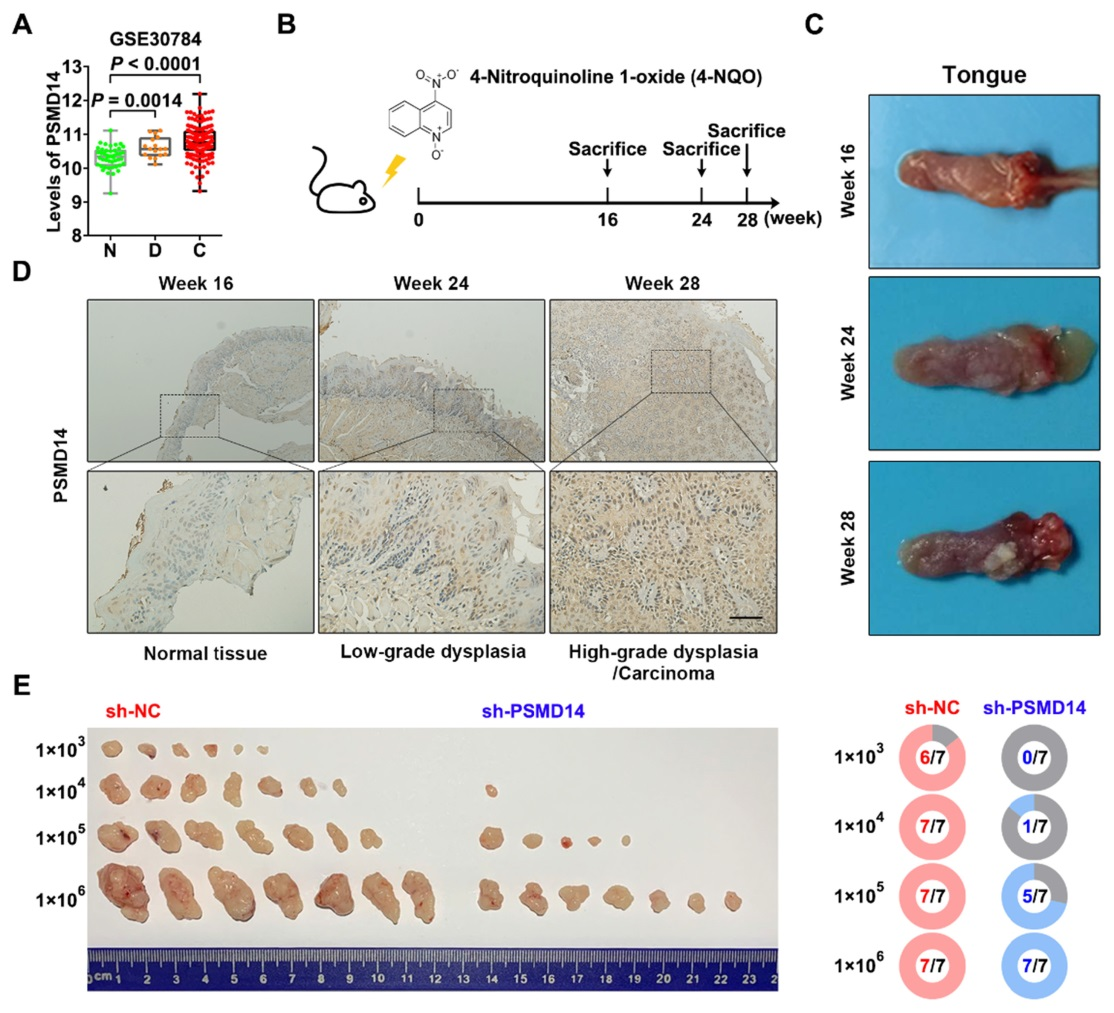
** PSMD14 promotes tumorigenesis of HNSCC. (A)** The mRNA expressions of *PSMD14* in normal tissue, dysplasia and HNSCC were shown by analyzing GEO dataset GSE30784. N, normal tissue. D, dysplasia. C, cancerous tissue. **(B)** A murine HNSCC model was established with the administration of chemical carcinogen 4-NQO (50μg/mL) dissolved in drinking water for 16 weeks. **(C)** The representative images of tongue specimens of mice collected at Week 16, 24 and 28, respectively. **(D)** The results of IHC showed that PSMD14 expression in dysplasia or HNSCC was higher than that in the normal tissues. Scale bar, 100 μm.** (E)** The result of limited dilution assay *in vivo* (7 mice per group) showed that PSMD14 depletion dramatically impaired tumorigenesis of HNSCC.

Interestingly, the analysis of another GEO dataset GSE30784 showed that the mRNA level of *PSMD14* was gradually increased from normal tissue to dysplasia to HNSCC (Figure 2A). Then, we established a 4-NQO-induced murine HNSCC model (Figure 2B), and collected the tongue specimens of mice at Week 16, 24 and 28 respectively (Figure 2C). The results of IHC confirmed that the protein expression of PSMD14 was much higher in low-grade dysplasia (Week 24) and high-grade dysplasia/carcinoma (Week 28) than that in normal tissue (Week 16) (Figure 2D), indicating that PSMD14 may be implicated in HNSCC tumorigenesis. To further validate the role of PSMD14 in the tumorigenic potential of HNSCC cells, a limiting dilution analysis was performed using SCC15 cells expressing control or PSMD14 shRNA. When the control SCC15 cells reached a tumor formation rate of 100% (7/7), even at a transplantation number of 1×10^4^ cells per mouse, PSMD14-depleted SCC15 cells reached a tumor formation rate of only 14.3% (1/7) at the same dilution rate (Figure 2E). These evidences illustrate that PSMD14 depletion impairs HNSCC initiation.

### Suppression of PSMD14 undermines proliferation and chemoresistance of HNSCC cells

**Figure 3:**
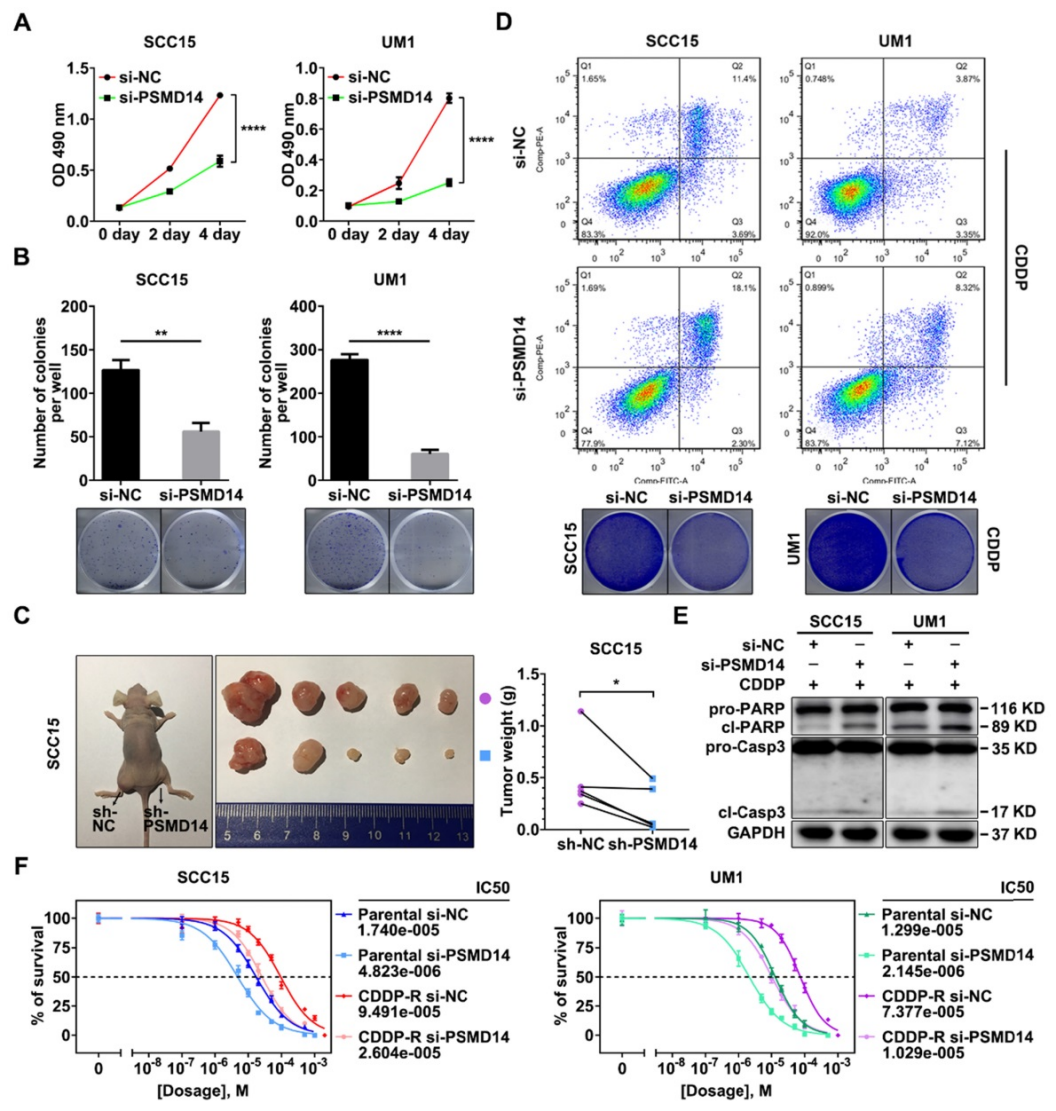
** Depletion of PSMD14 inhibits tumor growth and sensitizes HNSCC cells to cisplatin. (A)** PSMD14 knockdown by specific siRNAs suppressed the growth of SCC15 and UM1 cells *in vitro*. Bar, SD for quintuplicate wells. **(B)** PSMD14 depletion significantly attenuated the capacity of colony formation of HNSCC cells.** (C)** The representative photos (left panel) and the weight (right panel) of xenografts of negative control and PSMD14-depleted group (n = 5). Reduced PSMD14 significantly inhibited the *in vivo* growth of HNSCC cells. **(D)** Depletion of PSMD14 promoted cisplatin-induced apoptosis, which was confirmed by flow cytometry (upper panel) and clonogenicity assay (lower panel). **(E)** The abundance of PARP and cleaved Caspase-3 was measured by immunoblotting in si-PSMD14-transfected HNSCC cells exposed to cisplatin. **(F)** The parental and CDDP-R HNSCC cells transfected with siPSMD14 were exposed to CDDP at various concentrations for 24 hours. Then, the IC50 values of CDDP in indicated groups were measured by using MTT assay. Data in this figure, mean ± SD, *P < 0.05, **P < 0.01, ****P < 0.0001. CDDP, cisplatin. CDDP-R, cisplatin-resistant.

We next explored the role of PSMD14 in HNSCC progression. A pool of three siRNAs was used to silence the expression of PSMD14 (Figure S1B). In view of the positive correlation between PSMD14 expression and T stage which reflects the malignant growth of HNSCC, we performed MTT assay, colony formation and *in vivo* growth assays to assess the effect of PSMD14 on cell proliferation. As shown, PSMD14 depletion significantly impeded the growth rate of HNSCC cells *in vitro* (Figure 3A). Besides, the capacity of colony formation was attenuated in the PSMD14-silenced HNSCC cells (Figure 3B). Furthermore, stable reduction in PSMD14 (Figure S1C) resulted in a considerable decrease of size and weight of xenografts (Figure 3C).

For another, the higher expression of PSMD14 in chemoresistant tissues indicated that PSMD14 may play a critical role in HNSCC chemoresistance. Therefore, the function of PSMD14 in chemoresistance was detected. At first, we measured the IC50 values of CDDP in HNSCC cells with distinct PSMD14 expression. The results revealed that CAL27 cell line was more sensitive to CDDP than SCC15 and UM1 cell lines with higher expression of PSMD14 (Figure S1A and Figure S2). Then, we established Cisplatin-resistant (CDDP-R) subclones in SCC15 and UM1 cell lines respectively, and identified elevated PSMD14 in CDDP-R HNSCC cells relative to the parental ones (Figure S3). According to these data, we supposed that PSMD14 was associated with intrinsic and acquired resistance to CDDP in HNSCC. Further, we found that PSMD14 knockdown dramatically sensitized HNSCC cells to CDDP by using flow cytometry and clonogenicity assay (Figure 3D and Figure S4), which was further confirmed by the detection of cleaved PARP and cleaved Caspase-3 (Figure 3E). In addition to HNSCC parental cells, CDDP-R cells also became sensitive to CDDP once PSMD14 was silenced (Figure 3F). Collectively, the above results convincingly demonstrate that PSMD14 heightens proliferation and chemoresistance to promote HNSCC progression.

### PSMD14 knockdown impairs E2F1/Akt/SOX2 axis-mediated stemness in HNSCC

**Figure 4:**
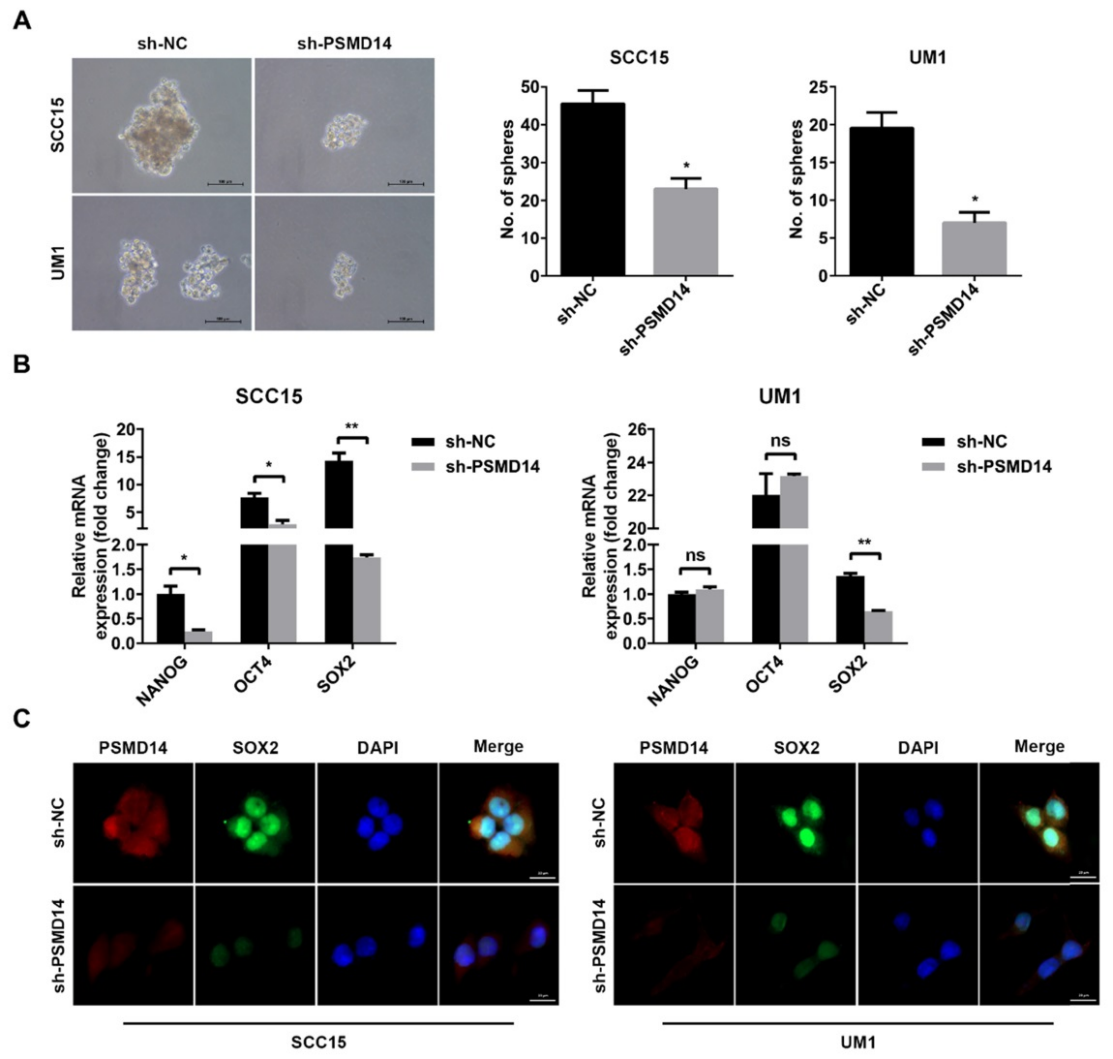
** PSMD14 silencing antagonizes cell stemness in HNSCC. (A)** The sphere formation ability was significantly reduced in shPSMD14-transfected HNSCC cells. Scale bar, 100 μm. **(B)** The mRNA level of cancer stem cells markers was measured in SCC15 and UM1 cells with stable infected PSMD14 knockdown. **(C)** The representative images of immunofluorescence staining of PSMD14 and SOX2 in stable PSMD14-depleted HNSCC cells. Scale bar, 20 μm. Data in **(A)** and **(B)**, mean ± SD, *P < 0.05, **P < 0.01, ns, no significance.

Tumor stemness is a major contributor to tumorigenesis and chemoresistance 23, 24, and even confers recurrence and poor prognosis 25, which has been proven to correlate with increased PSMD14. Hence, we assessed whether PSMD14 functioned as an oncogene by regulating stemness in HNSCC. As shown in Figure 4A, the size and number of spheres were significantly reduced in PSMD14-deficient HNSCC cells compared with the control cells. Then, we further detected the expression of stemness markers including NANOG, OCT4 and SOX2. The outcomes of qPCR showed that SOX2 was decreased as the result of reduced PSMD14 level in both SCC15 and UM1 cells (Figure 4B), which was confirmed by the staining of immunofluorescence (Figure 4C).

**Figure 5:**
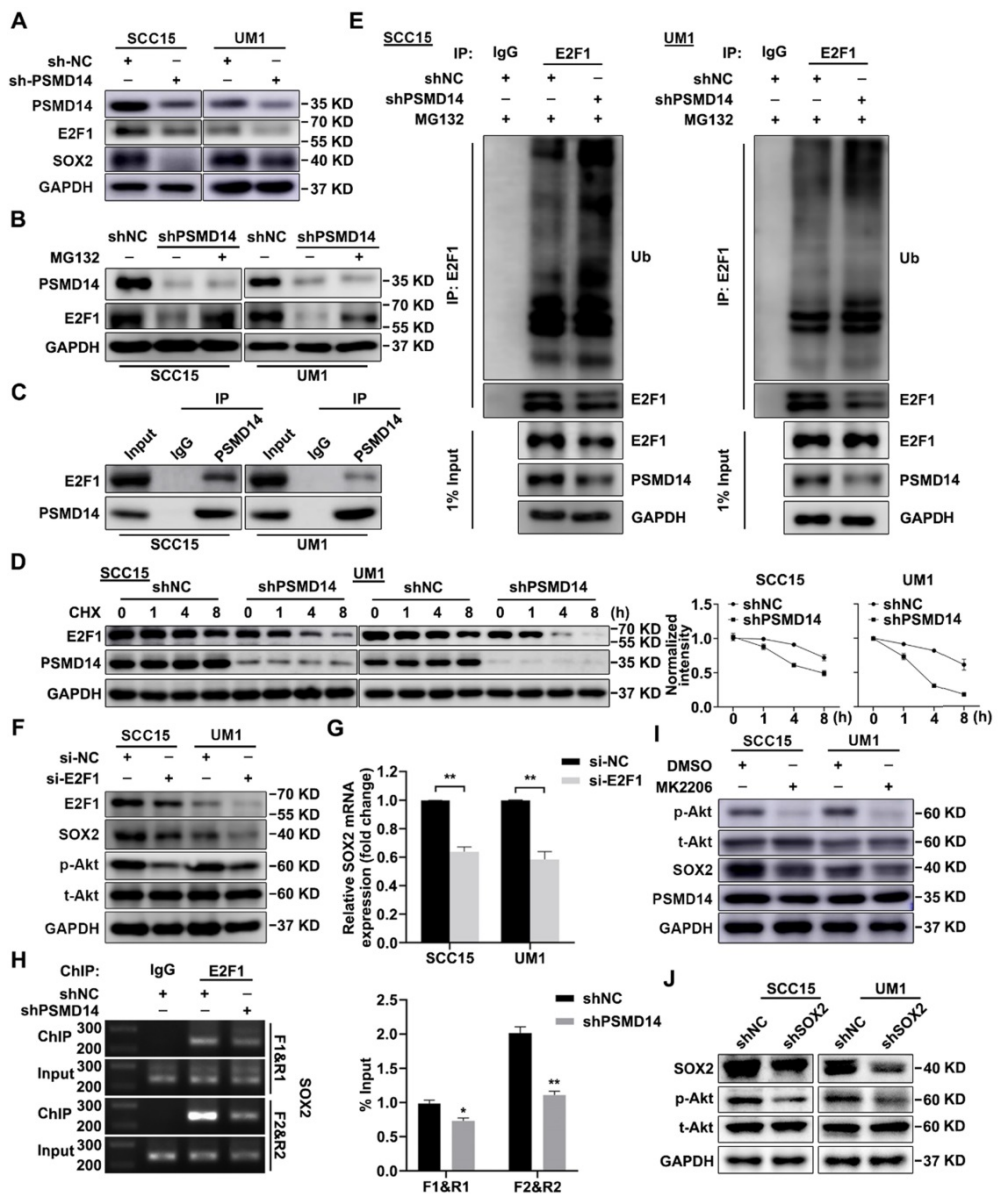
** PSMD14 stabilizes E2F1 to strengthen Akt/SOX2 axis. (A)** The abundance of PSMD14, E2F1 and SOX2 was probed in the shNC and PSMD14 knockdown HNSCC cells. **(B)** The immunoblotting assay showed that MG132 mitigated the inhibition of PSMD14 on E2F1. **(C)** Endogenous PSMD14 proteins were immunoprecipitated with anti-PSMD14 antibody or IgG, and then analyzed by immunoblotting assay. **(D)** SCC15 and UM1 cells expressing PSMD14 shRNA were exposed to 0.05 mg/ml CHX at the indicated time point for 0, 1, 4, 8 hours. The E2F1 protein expression was analyzed by immunoblotting assay and quantified by ImageJ software. **(E)** The poly-ubiquitination level of endogenous E2F1 in HNSCC cells stably transfected with shNC or shPSMD14 was assessed by *in vivo* ubiquitination assay. 1% input of cell lysates was used to assess the expression of E2F1 and PSMD14. **(F)** The abundance of E2F1, SOX2 and p-Akt was measured in SCC15 and UM1 cells transiently transfected with si-E2F1. **(G)** The results of qPCR revealed that si-E2F1 reduced the mRNA expression of SOX2. **(H)** PSMD14 depletion significantly decreased the binding of E2F1 to SOX2 promoter. **(I)** The protein expression of PSMD14, p-Akt and SOX2 was detected in SCC15 and UM1 cells treated with MK2206. **(J)** The abundance of SOX2 and p-Akt was measured in SCC15 and UM1 cells expressing SOX2 shRNA. Data in **(D)**, **(G)** and **(H)**, mean ± SD, *P < 0.05, **P < 0.01.

It is reported that transcription factor E2F1 confers the maintenance of cell stemness 26 and affects the expression of SOX2 27. Through immunoblotting assay, we confirmed that PSMD14 blocking inhibited the expression of E2F1 (Figure 5A), while no obvious change was found in E2F1 mRNA level (Figure S5). Furthermore, the inhibition was nearly abrogated by the proteasome inhibitor MG132 (Figure 5B). As shown in Figure 5C, the interaction between endogenous PSMD14 and E2F1 in HNSCC was verified by immunoprecipitation assay. Additionally, the results of CHX pulse-chase assay showed that PSMD14 knockdown shortened the half-life of E2F1 and accelerated its degradation (Figure 5D). Then, we validated that the ubiquitination level of E2F1 was greatly elevated in HNSCC cells stably transfected with shPSMD14 (Figure 5E), indicating that PSMD14 prevented the degradation of E2F1 through ubiquitin-proteasome system (UPS). Notably, recent studies have discovered that CDDP-resistant HNSCC cells are characterized by the increased activity of Akt pathway 28, which drives cancer cell stemness in esophageal squamous cell carcinoma (ESCC) 29. In our study, we found that Akt was continuously phosphorylated in HNSCC cells exposed to CDDP (Figure S6), illustrating the potential role of Akt activation in antiapoptotic process of HNSCC. Interestingly, PSMD14 silence also dramatically inhibited the phosphorylation of Akt, but the activation of other antiapoptotic pathways such as Erk1/2 and Stat3 signaling was not affected (Figure S7). When HNSCC cells were transfected with specific siRNAs targeting E2F1, the downstream target of PSMD14, both SOX2 and p-Akt was inhibited (Figure 5F). The results of qPCR showed that the mRNA level of SOX2 was also decreased in si-E2F1 transfected cells (Figure 5G). Furthermore, PSMD14 knockdown hindered the E2F1-stimulated transcription of SOX2 (Figure 5H). Additionally, Akt inhibitor MK2206, resembling si-PSMD14 and si-E2F1, obviously attenuated SOX2 expression (Figure 5I). In turn, the activation of Akt was inhibited by SOX2 depletion (Figure 5J). Briefly, PSMD14 suppressed the degradation of E2F1, and then E2F1 promoted AKT/SOX2 positive feedback loop.

**Figure 6:**
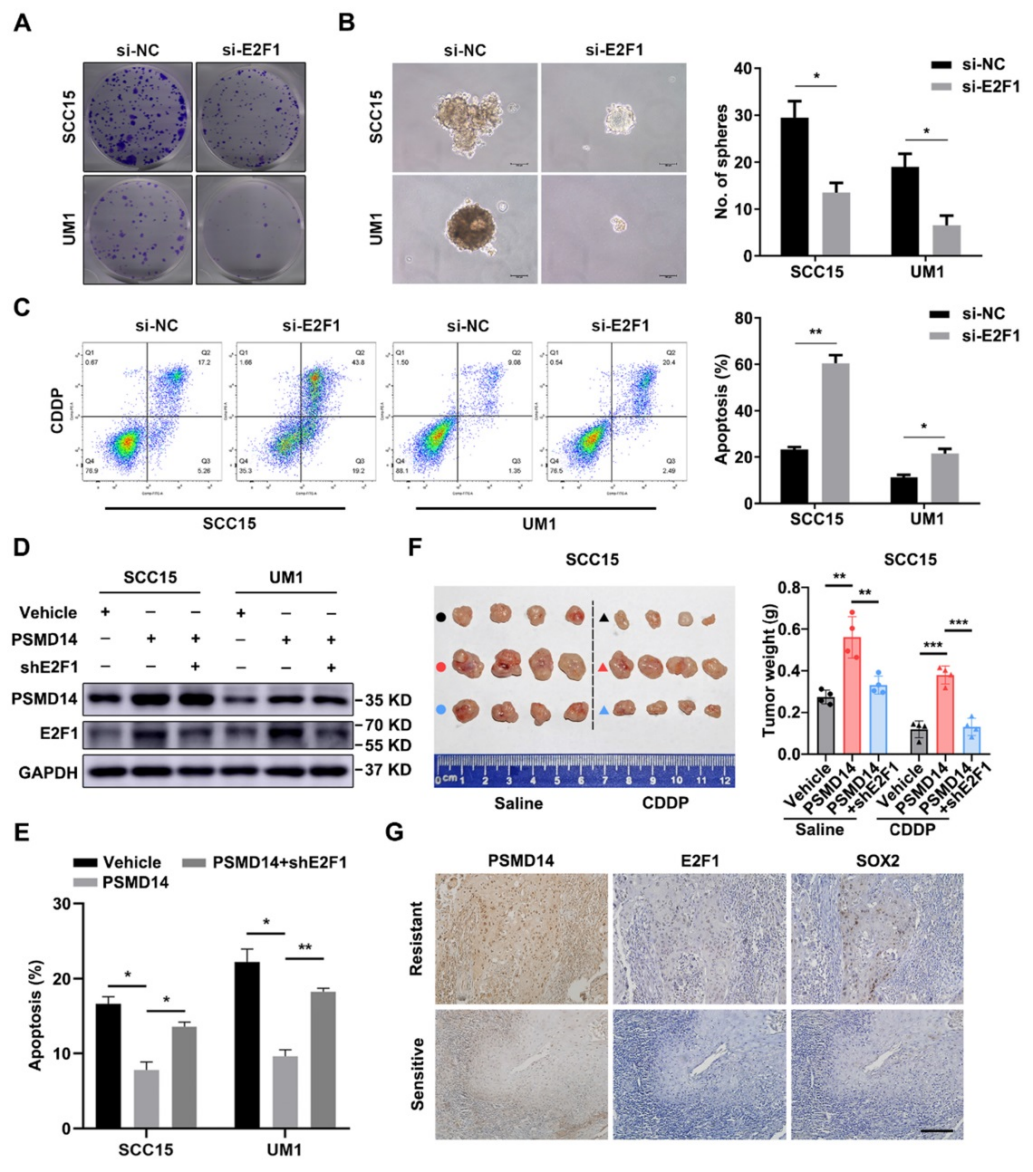
** PSMD14 promotes malignant progression of HNSCC by regulating E2F1. (A)** The colony formation ability in E2F1-depleted HNSCC cells was measured. Representative images were shown. **(B)** Depletion of E2F1 significantly impaired the capacity of sphere formation in SCC15 and UM1 cells. Scale bar, 100 μm. **(C)** The cisplatin-induced apoptosis of si-E2F1 transfected HNSCC cells was detected by flow cytometry. **(D)** The abundance of PSMD14 and E2F1 was measured in indicated groups. **(E)** The statistical histogram of flow cytometry showed that compared with the control group, PSMD14 overexpression significantly improved the resistance of HNSCC cells to CDDP, while E2F1 depletion re-sensitized HNSCC cells expressing PSMD14 to CDDP. **(F)** Tumor formation in BALB/c nude mice (n = 4) injected subcutaneously with SCC15 cells as indicated. The photograph of the xenografts in each group was shown and the tumor weight was quantified. **(G)** The levels of PSMD14, E2F1 and SOX2 in sensitive and resistant HNSCC tissues were measured by using IHC staining. Scale bar, 100 μm. Data in this figure, mean ± SD, *P < 0.05, **P < 0.01, ***P < 0.001. CDDP, cisplatin.

To further verify the importance of E2F1 and Akt signaling in HNSCC progression, a series of functional experiments were conducted. As shown in Figure 6A and 6B, si-E2F1 significantly weakened the capacities of colony formation and spheroid formation. Besides, knockdown of E2F1 sensitized both HNSCC cell lines to the treatment of CDDP (Figure 6C). Similar to si-E2F1, MK2206 also undermined the colony formation, stemness and chemoresistance of HNSCC cells (Figure S8A-C). Then, we found that PSMD14 overexpression elevated the level of E2F1 (Figure 6D) and decreased CDDP-induced apoptosis *in vitro* (Figure 6E and Figure S9), while E2F1 silencing circumvented the resistance to CDDP mediated by PSMD14 (Figure 6E and Figure S9). Inversely, PSMD14 depletion weakened E2F1 expression and promoted the apoptosis of SCC15 and UM1 cells treated with CDDP (Figure S10A-B). Then, E2F1 re-expression rescued shPSMD14-impaired chemoresistance of HNSCC cells (Figure S10A-B). Furthermore, the results of *in vivo* assay showed that the xenografts generated from PSMD14-overexpressing HNSCC cells weighed significantly more than that generated from negative control cells, and showed a less decrease in tumor weight compared with the control group under the same treatment dose of CDDP (Figure 6F). Conversely, the tumor-promoting activity of PSMD14 *in vivo* was counteracted by the stable knockdown of E2F1 (Figure 6F). Moreover, the PSMD14, E2F1 and SOX2 levels were highly upregulated in the chemoresistant clinical samples and chemoresistant HNSCC cells (Figure 6G and Figure S3). Together, these results corroborate that PSMD14 improves HNSCC chemoresistance by sustaining E2F1/Akt/SOX2 axis-mediated stemness.

### PSMD14 inhibitor Thiolutin exerts anti-tumor activity in HNSCC

**Figure 7:**
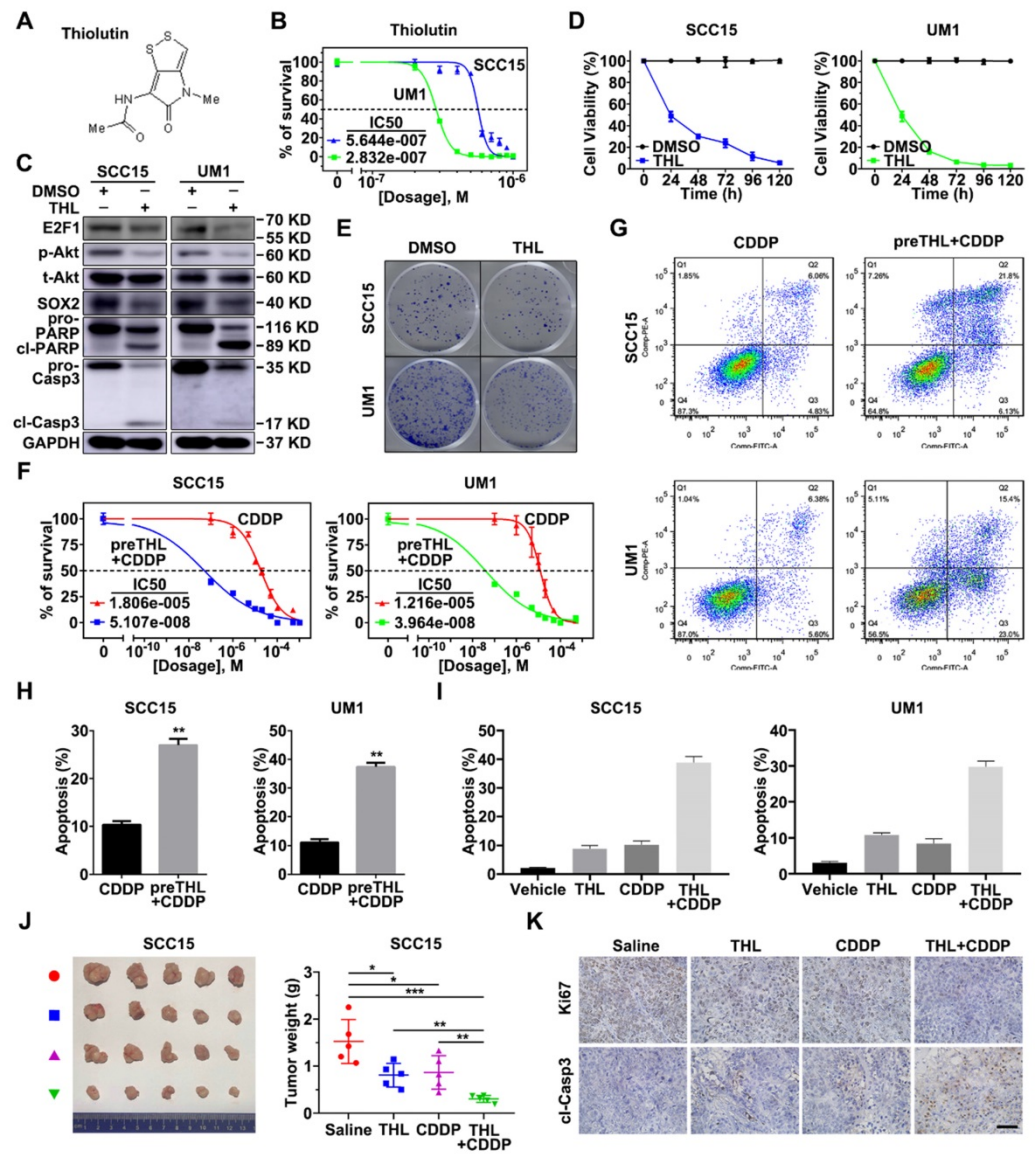
** PSMD14 inhibitor Thiolutin improves chemotherapy in HNSCC. (A)** The chemical structure of Thiolutin. **(B)** The IC50 of Thiolutin in SCC15 and UM1 cell lines was measured. **(C)** The abundance of E2F1/Akt/SOX2 axis modulated by PSMD14 and proapoptotic markers was probed in SCC15 and UM1 cells treated with Thiolutin for 24 hours. **(D)** The cell viability of HNSCC cells was extremely inhibited by Thiolutin treatment. **(E)** THL attenuated the colony formation of SCC15 and UM1 cells. **(F**-**H)** The measurement of cisplatin IC50 **(F)** and flow cytometry **(G** and **H)** showed that THL significantly increased sensitivity of HNSCC cells to cisplatin. **(I)** The apoptosis rates of HNSCC cells treated with THL, CDDP and THL plus CDDP were measured by using flow cytometry. The concentration of THL in **(C-I)** was 0.6 μM in SCC15 cells and 0.3 μM in UM1 cells, respectively. **(J)** The mice (n = 5) with xenografts were administered with saline, THL (0.75 mg/kg), CDDP (2.5 mg/kg) or CDDP (2.5 mg/kg) combined with THL (0.75 mg/kg) every 3 days until sacrifice. Then the representative photos of indicated treated HNSCC xenografts were presented. The size and weight of tumors were significantly reduced in combined treatment group. **(K)** The expression of Ki67 and cleaved Caspase-3 was measured by IHC staining among four groups described in** (J)**. Scale bar, 100 μm. Data in this figure, mean ± SD, *P < 0.05, **P < 0.01, ***P < 0.001. THL, Thiolutin. CDDP, cisplatin

To translate the bench results into clinical practice, the effect of novel small-molecule inhibitor of PSMD14 in HNSCC was further explored. We found that Thiolutin (THL, Figure 7A), a disulfide-containing antibiotic and anti-angiogenic compound 30, dramatically inhibited DUB activity of PSMD14 (compared with other JAMM DUBs) in a dose-manner dependent using Ubiquitin-AMC assay (Figure S11), which was in line with the results of previous studies 31. Then, we determined the IC50 of THL in SCC15 (IC50 = 0.5644 μM) and UM1 (IC50 = 0.2832 μM) cells (Figure 7B). The immunoblotting analysis showed that the administration of THL to HNSCC cells strikingly inactivated E2F1/Akt/SOX2 pathway and increased the activation of PARP and Caspase-3 (Figure 7C). As shown, cell viability and colony formation of SCC15 and UM1 cells were significantly impaired under THL treatment (Figure 7D-E). In addition, THL pretreatment extremely increased sensitivity of HNSCC cells to CDDP (Figure 7F), and promoted CDDP-induced apoptotic cascade (Figure 7G-H). The *in vitro* and *in vivo* experiments showed that compared with saline, THL or CDDP, the combined administration of CDDP and THL significantly facilitated the apoptosis of HNSCC cells (Figure 7I and Figure S12) and inhibited the growth of xenografts (Figure 7J), suggesting that THL improved the chemotherapeutic efficacy in HNSCC*.* Finally, IHC assay was performed to measure the expression of Ki67, a negative prognostic factor in HNSCC 32. As shown in Figure 7K, the positive rate of Ki67 in THL or CDDP-treated group was reduced relative to the control group, while the majority of tumor cells in combined treatment group were identified as Ki67 negative. Besides, the immunohistochemistry staining of cleaved Caspase-3 in combined treatment group was strongly positive compared with the other three groups (Figure 7K). Overall, these findings indicate that THL could serve as a promising adjuvant to increase chemosensitivity in HNSCC by targeting PSMD14.

## Discussion

Similar to the balance of phosphorylation by phosphatases, ubiquitination could be reversed by deubiquitinating enzymes (DUBs). Growing evidence show that the dysregulation of DUBs, such as mutation or altered expression, correlates with various human diseases, ranging from immune disease to cancer. For instance, the mutation of TNFAIP3, a negative regulator of NF-κB responses, results in an increase of NF-κB-mediated proinflammatory cytokines and autoinflammatory disease 33. The deletions or mutations of TNFAIP3 are found in about 30% of patients with diffuse large B-cell lymphoma, while reinforced TNFAIP3 induces apoptosis and cell growth arrest 34, suggesting TNFAIP3 is a tumor suppressor. Interestingly, the roles of DUBs are poles apart due to the specific context of diverse cancers. In breast cancer, DUB USP9X strongly interacts with LATS kinase to regulate Hippo pathway and suppress tumor growth 35. On the contrary, USP9X acts as an oncogene and stabilizes MCL1, a critical antiapoptotic member of the BCL-2 family, and thereby promotes cell survival of multiple myeloma 36. In view of the critical roles of DUBs in cancer progression, we thus supposed to screen the key DUBs in HNSCC. Among 90 candidates, ten DUBs (PSMD14, TNFAIP3, JOSD1, UFD1L, UCK2, SENP5, COPS5, USP5, USP31 and USP39) were found to be significantly increased in HNSCC using bioinformatics analysis. As one candidate, PSMD14 is one essential component of 26S proteasomal subunit, and removes the ubiquitin chain from targeted proteins to facilitate further degradation of substrates. In contrast, accumulating evidence reveal that PSMD14 also suppress the ubiquitination and degradation of specific proteins, such as SNAIL 37, GRB2 38 and ALK2 receptor 39, to promote cancer progression. Therefore, PSMD14 may perform like a “controller” of recycling station to proofread and determine the fate of substrates. In this study, we identified that PSMD14 was aberrantly expressed in the dysplasia and HNSCC compared to normal tissue using a 4-NQO-induced murine HNSCC model, and correlated positively with clinical stage, T stage and recurrence of patients with HNSCC. Furthermore, the increase of PSMD14 indicated poor prognosis in HNSCC. Loss-of-function assays showed that PSMD14 depletion undermined tumor initiation, growth and chemoresistance of HNSCC cells. These data suggest that PSMD14 acts as an oncogene to promote HNSCC progression, and is implicated in the tumorigenesis of HNSCC, which should be further determined using knockout mice with 4-NQO administration.

Compelling evidence has unraveled that stemness is a major contributor to tumorigenesis and chemoresistance, as well as recurrence 9, 40, which were robustly associated with PSMD14. As a key transcription factor, SOX2 confers tumor initiation and stemness in squamous cell carcinoma 41. We found that PSMD14 knockdown impaired stemness of HNSCC cells by inhibiting SOX2. Of note, the mRNA expression of SOX2 was decreased while PSMD14 functions at the post-translational level, indicating that PSMD14 regulates SOX2 expression indirectly and a potential “bridge” between them should exist. Importantly, Courtney M Schaal *et al.* has demonstrated that nicotine and electronic-cigarettes could stimulate the expression of SOX2 through E2F1 27, a critical transcription factor that participates in cancer malignant progression 42-44. Then, we pinpointed that PSMD14 inhibited ubiquitination and degradation of E2F1, which is consistent with the results reported previously 45. Further, the ChIP assay showed that E2F1 bound to the promoter of SOX2, which could be attenuated by PSMD14 silencing. Therefore, our data indicate that PSMD14-mediated increase of E2F1 directly promotes the transcription activation of SOX2. Besides, we also found that both PSMD14 and E2F1 conferred the activation of Akt signaling pathway that is associated with cancer cell survival and stemness 46, 47. The E2F1-mediated activation of Akt may be caused by the transcriptional regulation of E2F1 on a docking protein Gab2 48. When Gab2 becomes tyrosine phosphorylated, it recruits SH2 domain‑containing protein such as PI3K, and thus activates Akt and other signaling pathways 49. In the meanwhile, suppression of Akt pathway by MK2206 reduced SOX2 expression, probably resulting from the Akt-stimulated protection of SOX2 proteasomal degradation 29. Reversely, SOX2 knockdown weakened Akt activation, which was in line with the results of previous study 50, indicating that there is a positive feedback loop between Akt and SOX2. These data suggest a E2F1/Akt/SOX2 axis regulated by PSMD14. Noticeably, the observations showing that E2F1 is implicated in senescence and apoptosis may suggest a dual role of E2F1 in cancer 51. Through functional experiments, we identified that the growth, stemness and chemoresistance of HNSCC were enforced by E2F1, as well as Akt pathway. Additionally, the rescue assays revealed that PSMD14 exerted tumor-promoting activity in HNSCC by regulating E2F1. The aberrant expression of these molecules mentioned above in resistant HNSCC tissues and cells also reflected their oncogenic roles in HNSCC progression.

JAB1/MPN/Mov34 (JAMM) domain, which cleaves off conjugates of ubiquitin depending on Zn^2+^, is indispensable for PSMD14 DUB activity 52. Thiolutin (THL) has been reported to be a zinc chelator to inhibit PSMD14 with a minimum IC50 compared with other JAMM proteases 31. Minamiguchi *et al.* found that THL inhibited the adhesion of human umbilical vein endothelial cells (HUVECs) to vitronectin by reducing paxillin in HUVECs and suppressed tumor cell-induced angiogenesis* in vivo* 53. Besides, THL could inhibit endothelial cell adhesion by induction of Hsp27 phosphorylation and block both wound-driven and tumor-driven vascular outgrowths by utilizing an *ex vivo* model 54. In this study, we found that THL dramatically suppressed the DUB activity of PSMD14 (with little effect on other JAMM DUBs) and E2F1/Akt/SOX2 pathway, which spurred tremendous interests to exploit THL as an anti-tumor drug. The *in vitro* and *in vivo* experiments showed that THL undermined cell viability and potently increased the sensitivity of HNSCC cells to cisplatin, the standard chemotherapy regimen for HNSCC. Therefore, THL may improve chemotherapy efficacy and reduce side effects by reducing CDDP dosage to benefit patients. A point worth emphasizing is that THL and other small-molecule inhibitors 55-57 targeting PSMD14 should not be completely equated with proteasome inhibitor which is an effective therapy for patients with multiple myeloma 58, 59, because of the dual role of their target PSMD14 in determining substrates fate. In addition, proteasome inhibitors such as bortezomib and carfilzomib, may be difficult to distinguish from normal tissues in their dependence on the ubiquitin proteasome system, which results in the barrier in the treatment of solid tumors 60. PSMD14 inhibitor is, therefore, a safer and more effective candidate for tumor treatment with higher targeting capacity.

In conclusion, these data demonstrate that PSMD14 in highly expressed in HNSCC and associated with tumorigenesis and malignant progression of HNSCC. Elevated PSMD14 indicates unfavorable prognosis of patients with HNSCC. PSMD14 increases cell resistance to cisplatin in HNSCC by enforcing E2F1/Akt/SOX2 axis-mediated stemness. Additionally, targeting PSMD14 by small-molecule inhibitor Thiolutin robustly enhances chemosensitivity of HNSCC cells* in vitro* and* in vivo*. Our findings offer ample evidence to support PSMD14 as a novel and promising target for diagnosis and treatment of HNSCC.

## Supplementary Material

Supplementary figures and tables.

## Abbreviations

ACC: adrenocortical carcinoma
BLCA: bladder urothelial carcinoma
BRCA: breast invasive carcinoma
CAFs: carcinoma-associated fibroblasts
CDDP: cisplatin
CESC: cervical squamous cell carcinoma and endocervical adenocarcinoma
CHOL: cholangio carcinoma
CHX: cycloheximide
COAD: colon adenocarcinoma
DLBC: lymphoid neoplasm diffuse large b-cell lymphoma
DUBs: deubiquitinating enzymes
ESCA: esophageal carcinoma
GBM: glioblastoma multiforme
GEO: gene expression omnibus
HNSC: head and neck squamous cell carcinoma
HPV: human papilloma virus
IC50: 50% inhibitory concentration
KICH: kidney chromophobe
KIRC: kidney renal clear cell carcinoma
KIRP: kidney renal papillary cell carcinoma
LAML: Acute Myeloid Leukemia
LGG: Brain Lower Grade Glioma
LIHC: liver hepatocellular carcinoma
LUAD: lung adenocarcinoma
LUSC: lung squamous cell carcinoma
MESO: mesothelioma
OV: ovarian serous cystadenocarcinoma
PAAD: pancreatic adenocarcinoma
PCPG: pheochromocytoma and paraganglioma
PRAD: prostate adenocarcinoma
READ: rectum adenocarcinoma
SARC: sarcoma
SKCM: skin cutaneous melanoma
STAD: stomach adenocarcinoma
STR: short tandem repeat
TCGA: the cancer genome atlas
TGCT: testicular germ cell tumors
THCA: thyroid carcinoma
THL: thiolutin
THYM: thymoma
UCEC: uterine corpus endometrial carcinoma
UCS: uterine carcinosarcoma
UPS: ubiquitin proteasome system
UVM: uveal melanoma
4-NQO: 4-nitroquinoline 1-oxide
