# Supplementary material for: Blockade of deubiquitinating enzyme PSMD14 overcomes chemoresistance in head and neck squamous cell carcinoma by antagonizing E2F1/Akt/SOX2-mediated stemness
Source: Theranostics. 2021 Jan 1;11(6):2655–69. doi: 10.7150/thno.48375 (PMC7806466; doi:10.7150/thno.48375)
Supplement: Supplementary file 1 — Supplementary figures and tables. [file thnov11p2655s1.pdf]

Supplementary Tables

Table S1. List of siRNAs sequence

siRNA name	Sequence (5'-3')
si-PSMD14-1	GTGCTGGAGTTCCAATGGA
si-PSMD14-2	GTGTGGATATCAACACTCA
si-PSMD14-3	GTTGGATACTGTCGTATTT
si-E2F1-1	GAGACCTCTTCGACTGTGA
si-E2F1-2	CTATGAGACCTCACTGAAT
si-E2F1-3	GGGAGAAGTCACGCTATGA

Table S2. List of qPCR primers sequence

Primer name	Sequence (5'-3')
GAPDH	F: TGCACCACCAACTGCTTAGC
	R: GGCATGGACTGTGGTCATGAG
PSMD14	F: GGAGGAGGTATGCCTGGACT
	R: TTAACAGTGCCAGGGAAGAGA
NANOG	F: TCTTCCTGGTCCCCACAGTTT
	R: GCAAGAATAGTTCTCGGGATGAA
OCT4	F: CACCATCTGTCGCTTCGAGG
	R: AGGGTCTCCGATTTGCATATCT
SOX2	F: GCGGAGTGGAAACTTTTGTCC
	R: CGGGAAGCGTGTACTTATCCTT
E2F1	F: AGCGGCGCATCTATGACATC
	R: GTCAACCCCTCAAGCCGTC

Table S3. List of ChIP-PCR primers sequence

Primer name	Sequence (5'-3')
SOX2 promoter	F1: GAAAAGGCGTGTGGTGTGAC
	R1: GTTTCTAGCGACCAATCAGCG
	F2: GGGAGTGCTGTGGATGAGC
	R2: GTGGGTAAACAGCACTAAGACTACGTG

Supplementary Figures

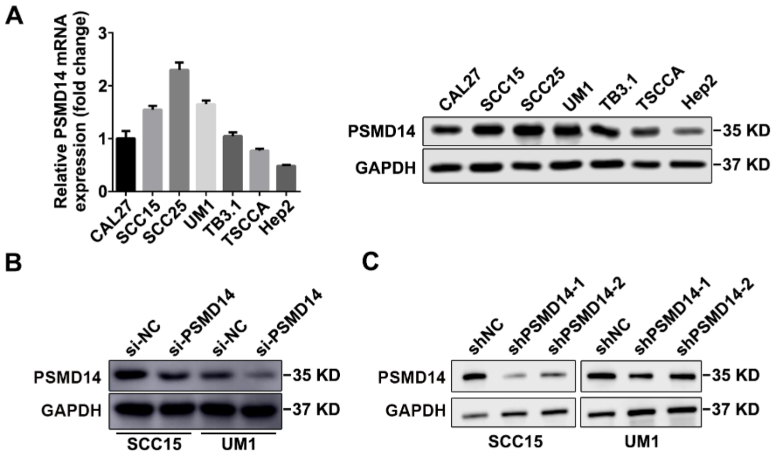

Figure S1. The level of PSMD14 in HNSCC cells. (A) The mRNA and protein expressions of PSMD14 were detected in a panel of HNSCC cell lines using qPCR (left panel) and immunoblotting assay (right panel). Data, mean ± SD. **(B)** The abundance of PSMD14 was probed in the HNSCC cells transfected with a pool of three siRNAs targeting PSMD14. **(C)** The protein expression of PSMD14 was measured in the HNSCC cells expressing control (shNC) and PSMD14 shRNAs (shPSMD14-1, shPSMD14-2).

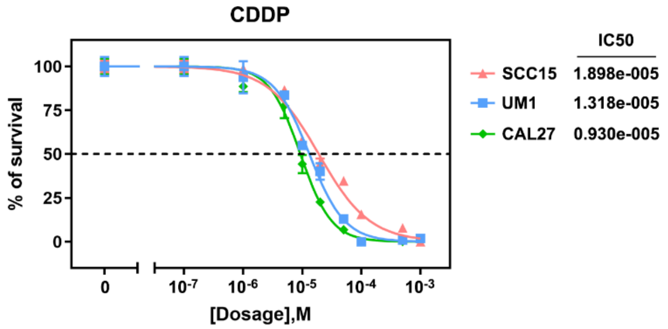

Figure S2. The IC_{50} value of cisplatin in HNSCC cell lines. Three HNSCC cell lines were exposed to cisplatin at various concentrations for 24 hours, and then MTT assay was conducted to calculate IC_{50} of cisplatin in these cell lines. CDDP, cisplatin.

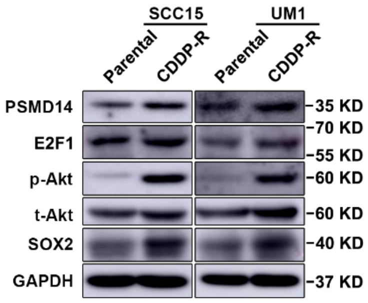

Figure S3. PSMD14/E2F1/Akt/SOX2 axis is aberrantly activated in cisplatin-resistant HNSCC subclones. The results of immunoblotting assay showed that the expressions of PSMD14, E2F1, p-Akt and SOX2 were all elevated in the CDDP-R cells compared with the parental HNSCC cells. CDDP-R, cisplatin-resistant.

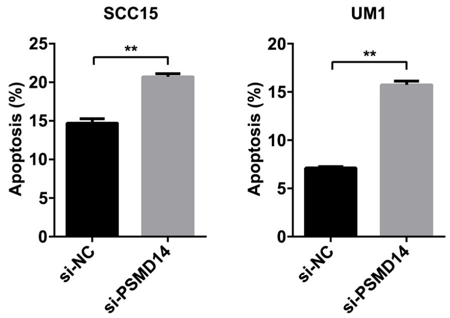

Figure S4. PSMD14 knockdown promotes CDDP-induced apoptosis of HNSCC cells. The statistical histogram showed that the apoptosis rate was significantly increased in PSMD14-silenced HNSCC cells under the treatment of cisplatin for 24 hours. Data, mean ± SD, $^{**}P < 0.01$.

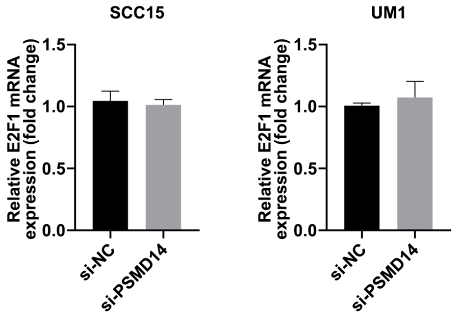

Figure S5. The mRNA expression of E2F1 is not obviously affected by PSMD14 depletion. The qPCR assay was performed to detect the expression of E2F1 mRNA in PSMD14-depleted HNSCC cells. Data, mean ± SD.

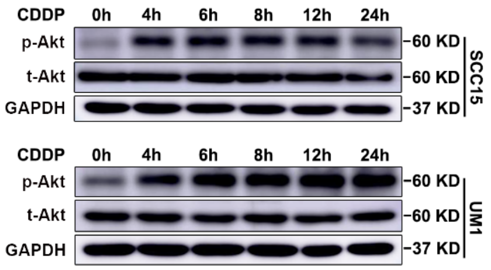

Figure S6. CDDP treatment activates Akt signaling pathway in HNSCC cells. The phosphorylation of Akt was detected under cisplatin treatment for indicated time. CDDP, cisplatin.

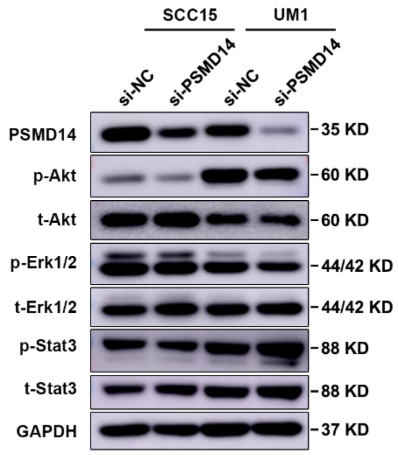

Figure S7. Depletion of PSMD14 inactivates Akt pathway. The immunoblotting assay was conducted to detect the activities of Akt, Erk1/2, Stat3 signaling pathways in the si-PSMD14-transfected HNSCC cells.

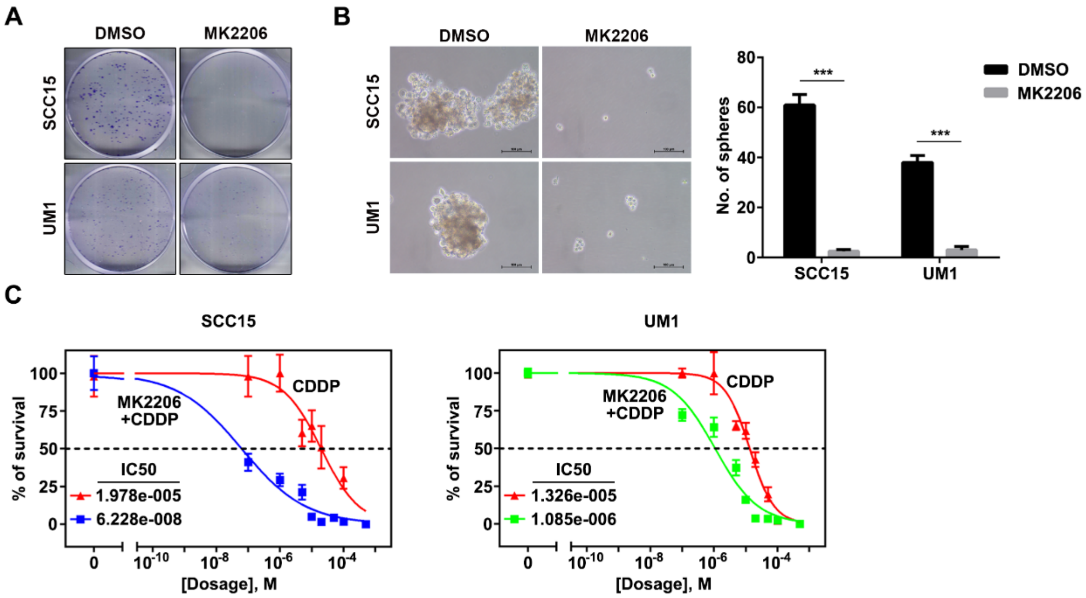

Figure S8. Akt inhibitor MK2206 exerts anti-tumor activities in HNSCC. (A) MK2206 weakened the colony formation of SCC15 and UM1 cells. **(B)** The size and number of spheres were both dramatically reduced with the exposure of MK2206. Scale bar, 100 μm. **(C)** MK2206 robustly lowered the IC_{50} value of cisplatin in both SCC15 and UM1 cell lines. Data in this figure, mean ± SD, ****P* < 0.001. CDDP, cisplatin.

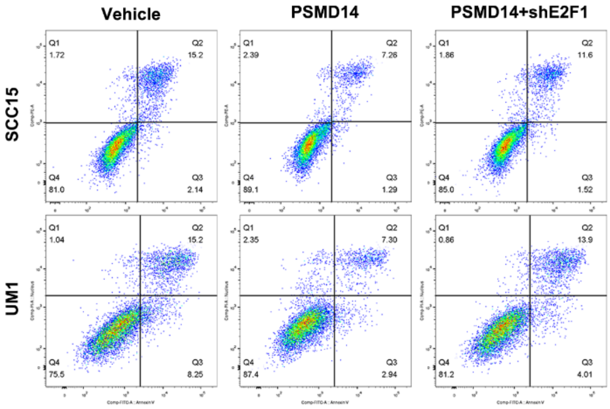

Figure S9. E2F1 depletion promotes cisplatin-induced apoptosis of HNSCC cells expressing PSMD14. The indicated groups were treated with cisplatin for 24 hours, then the cell apoptosis was detected by using flow cytometry.

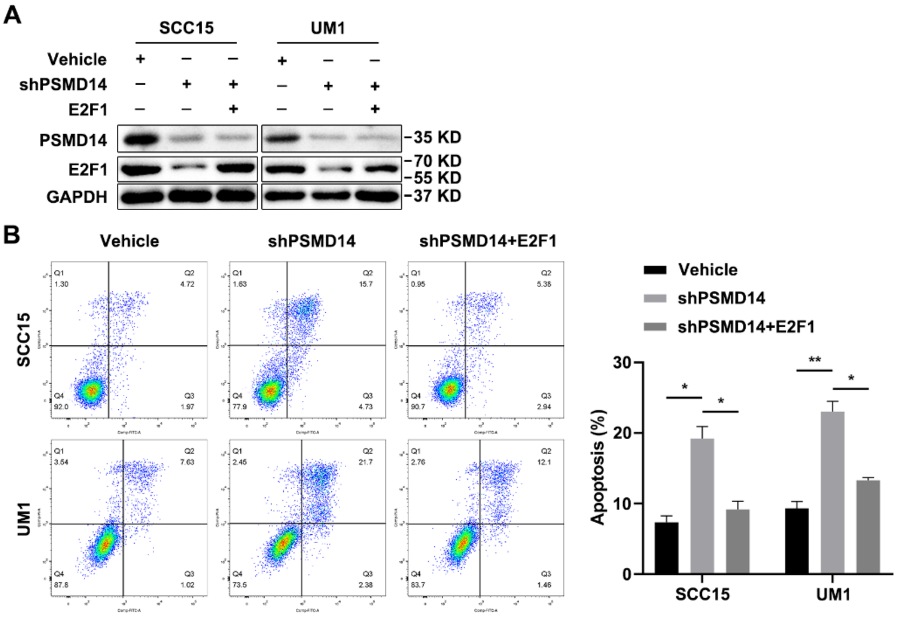

Figure S10. E2F1 strengthens chemoresistance of HNSCC cells expressing shPSMD14. (A) The protein expression of PSMD14 and E2F1 was measured by using immunoblotting in indicated groups. **(B)** The indicated groups were exposed to cisplatin for 24 hours, followed by apoptosis detection by using flow cytometry.

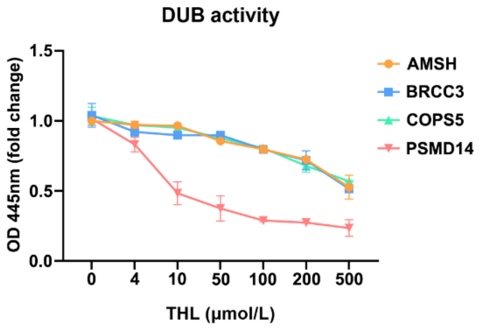

Figure S11. Thiolutin suppresses deubiquitinating enzyme activity of PSMD14 in a dose-dependent manner. Recombinant human DUBs belonging to JAMM family (PSMD14, AMSH, BRCC3 and COPS5) were incubated with THL respectively, then the absorbance at OD 445 nm was measured to detect DUB activity using Ubiquitin-AMC assay. Data, mean ± SD. THL, Thiolutin.

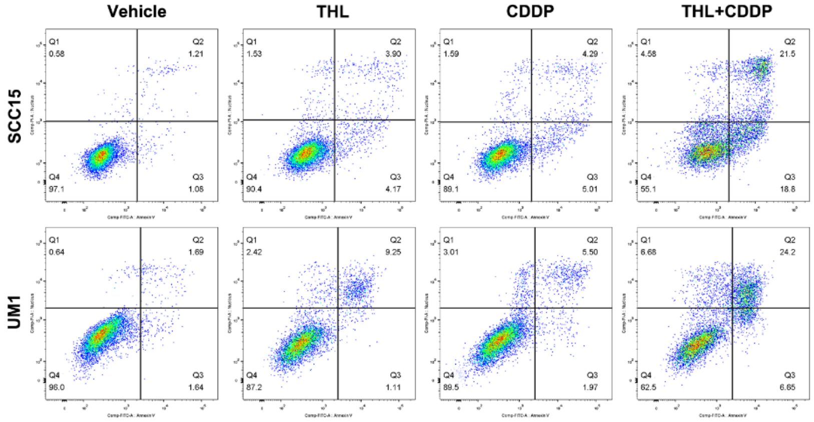

Figure S12. The combined treatment of CDDP and THL promotes apoptosis of HNSCC cells. The HNSCC cells were treated with THL, CDDP or THL plus CDDP for 24 hours respectively. Then, the apoptosis was detected by using flow cytometry. THL, thiolutin. CDDP, cisplatin.
